# The role of immunohistochemical tests in multimodal treatment of the aggressive forms of breast cancer

**Published:** 2014

**Authors:** D Mihai, S Voiculescu, C Dimitriu, F Constantinescu, S Stanilescu, T Burcos

**Affiliations:** * Surgery Clinic, “Coltea” Hospital, Bucharest

**Keywords:** receptors for estrogen, progesterone, HER2, cathepsin D, invasive form

## Abstract

Aggressive breast cancer is an invasive form with a differentiation degree G3/G4, the absence of estrogen receptor and progesterone and the absence or presence of the gene HER 2(+ or 3+). The immunohistochemical tests have an important role in establishing the diagnosis and the therapy.

**Material and method:** It was shown that the aggressive breast cancers, 97 out of 316 cases were operated in the period October 2011 - February 2014. The criteria of inclusion/ exclusion in the study groups and the treatment schemes were exposed.

**Results:** For the study group (group A=43/ group B=45/ group C=9 cases), the distribution according to the age group and immunohistochemical classification, were shown and, histologically, the type of surgical intervention, postoperative staging, postoperative complications were highlighted.

**Conclusions:** The treatment of the aggressive forms of breast cancer, neoadjuvant and adjuvant can both be set only by IHC tests.

## Introduction

Aggressive breast cancer is the leading cause of morbidity and mortality from cancer of the female population from Romania. The number of aggressive forms of breast cancer has increased in recent years, an important role in this issue being the one of the environmental factors, endogenous as well as genetics. The objective of the study is to determine the most effective therapeutic strategy for the aggressive breast cancer by using IHC tests.

## Material and method

316 patients with breast cancer were hospitalized and operated in “Coltea” Surgery Clinic, over a period of 29 months (October 2011 - February 2014), of whom 97 patients - 30.69% (94 women and 3 men) presented aggressive forms of cancer.

The diagnosis of aggressive breast cancer for the study group was established preoperatively by cumulating the clinical, paraclinical, histopathological and immunohistochemical diagnosis.

Inclusion criteria: breast cancers diagnosed by biopsy or tumor excision, which presented the clinical characteristics, histopathological and immunohistochemical forms of aggression.

The exclusion criteria were the following: breast cancers which occurred in pregnancy or when other pathologies were present.

The 97 patients were divided into 3 groups:

● Group A, made up of 43 patients (42 women + 1 man) had not received the neoadjuvant treatment.

● Group B, made up of 45 patients (44 women + 1 man) had received the PCT neoadjuvant treatment.

● Group C, made up of 9 patients (8 women + 1 man) have benefited from the PCT + RT/ RT neoadjuvant treatment.

The distribution according to the age group for each lot is presented in **Table 1**.

**Table 1:** Distribution according to the age group

	21-30	31-40	41-50	51-60	61-70	71-80	81-90
Group A	0	4	3	12	12	12	0
Group B	0	3	4	20	13	5	0
Group C	1	0	1	4	0	1	2
	1.03%	7.12%	8.21%	37.11%	25.77%	18.55%	2.06%

## Results

Many parameters such as the following were used in the study group: 

● From the histological point of view, the following forms of cancer were diagnosed: 

→CDI – 53 cases (54.63%)

→CLI – 9 cases (9.27%)

→CDI + CLI – 16 cases (16.49%)

→C metaplastic – 11 cases (11.34%)

→CDI + other forms – 8 cases (8.24%)

The histopathological result for each group of patients is presented in **Table 2**.

**Table 2:** Histopathological results

	CDI	CLI	CDI + CLI	C. Metaplastic	CDI + other forms
Group A	27	2	7	3	4
Group B	22	5	8	7	3
Group C	4	2	1	1	1
	54.63%	9.27%	16.49%	11.34%	82.24%

From the immunohistochemical point of view, the results were the following:

ER(-)/ PR(-)/ HER(-) – 79 cases = 81.44%

ER(-)/ PR(-)/ HER(+) – 15 cases = 15.46%

ER(-)/ PR(-)/ HER(+++) - 3 cases = 3.57%

The immunohistochemical result for each group of patients is presented in **Table 3**.

**Table 3:** IHC results

	ER(-), PGR(-), HER2(-)	ER(-), PGR(-), HER2(+)	ER(-), PGR(-), HER2(+++)
Group A	37	5	1
Group B	35	8	2
Group C	7	2	0
	81.44%	15.46%	3.09%

From the etiological point of view, each of the three types of risk factors had an important role:

- environmental factors: breast trauma, stress, smoking - 7 cases (11.29%).

- endogenous factors: nulliparity, lack of breastfeeding, breast benign tumors, endocrine disorders, obesity - 41 cases (66.12%).

- genetic factors: mutations of the BRCA1, BRCA2 genes and the familial aggregation Syndrome - 14 cases (22.58%).

 ● In terms of symptoms, all 97 patients presented the following characteristics, depending on the stage of the disease: pain, axillary lymphadenopathy, skin retraction.

● Postoperative pTNM staging was as it follows: 

→stage I: 11 cases (11.34%), 

→stage II: 40 cases (41.23%),

→stage III: 17 cases (17.52%),

→stage IV: 29 cases (29.89%).

The staging for each group of patients is presented in **Table 4**.

**Table 4:** Postoperative stadialization

	ST. I	ST. II	ST. III	ST. IV
Group A	8	18	5	12
Group B	3	19	9	14
Group C	0	3	3	3
	11.34%	41.23%	17.52%	29.89%

The surgical interventions performed were the following: 

- radical mastectomy Madden type: 82 cases (84.53%),

- simple mastectomy: 2 cases (2.06%),

- simple mastectomy + axillary lymphadenectomy: 2 cases (2.06%),

- sectorectomy + axillary lymphadenectomy: 9 cases (9.27%),

- simple mastectomy + partial resection of the pectoral muscles: 1 case (1.03%),

- Madden mastectomy + partial resection of the pectoral muscles invaded: 1 case (1.03%).

The surgical interventions for the 3 groups of patients are presented in **Table 5**.

**Table 5:** Types of surgeries

	Group A	Group B	Group C
Radical mastectomy Madden type (84.53%)	32	41	9
Simple mastectomy (2.06%)	2	0	0
Simple mastectomy + axillary lymphadenectomy (2.06%)	2	0	0
Sectorectomy + axillary lymphadenectomy (9.27%)	7	2	0
Simple mastectomy + partial resection of the pectoral muscles (1.03%)	0	1	0
Radical mastectomy Madden type + partial resection of the pectoral muscles (1.03%)	0	1	0

The main therapy is surgery cumulated with the PCT, RT, modern therapy targeted performed as neoadjuvant or adjuvant treatment.

● Postoperatory, the evolution was favorable, the main complication being lymphology, determined by the presence of axillary adenopathy, present in 59 cases, as it follows:

- stage I: 6 cases (10.16%)

- stage II: 14 cases (23.72%)

- stage III: 14 cases (23.72%)

- stage IV: 25 cases (42.37%)

Mortality and morbidity rates were 0.

## Discussion

Aggressive breast cancer is an invasive form with a G3/ G4 differentiation degree, absence of the receptors for estrogen, progesterone and the presence or absence of HER2 gene.

An important role in breast cancer was the one of the genetic factors along with the environment and the endogenous characteristics.

The genetic factors involved:

a) direct inheritance of the specific genetic defects, such as mutations of the BRCA1 gene which occurs in chromosome 17 [**1**].

b) modified transmission of BRCA2 gene competes in the occurrence of the breast cancer and especially in ovarian cancer [**1**].

Lynch [**2**] described the presence of several cancers of colon, stomach and breast in the same family members.

Studies have shown that the mutation carriers of BRCA1 gene presence in about 90% of ER (-), have a particular profile of gene expression. Certain anatomopathological features can help in the identification of breast tumors with mutations in the BRCA1 gene (cancers with a high degree of differentiation, increased mitotic index, lymphocytic infiltrate without HER2 receptor and without estrogen receptors); women carrying BRCA1 gene mutations, those with ER (-) or those with a history of breast cancer without estrogen receptors have an increased risk of developing a tumor ER (-) [**3**].

The role of genetic factors in the occurrence of mammary cancer in the three study groups is presented as it follows:

Group A - breast neoplasm (4 cases), gastric neoplasm (1 case), colon neoplasm (1 case), genital neoplasm (1 case).

Group B - breast neoplasm (3 cases), gastric neoplasm (1 case), colon neoplasm (1 case), genital neoplasm (1 case).

Group C - breast neoplasm (1 case), gastric neoplasm (1 case).

The clinical and paraclinical diagnosis cumulated establish the tumor location, axillary adenopathy presence, nipples discharge.

Laboratory diagnosis is supported by CA 15-3, TAG 72, MCA ACE antigens and cathepsins D.

The level of tumor markers was the following for the three groups of patients:

→ Group A: 6 cases stage I, stage II, 9 cases, 5 cases of stage III, 11 stage IV cases.

→ Group B: 1 case of stage I, stage II 13 cases, 8 cases of stage III, 14 stage IV cases.

→ Group C: 2 cases of stage II, stage III 3cazuri 4 cases, stage IV.

The accurate diagnosis is obtained by cumulating the histopathological diagnosis and also the immunohistochemical one.

The histopathological diagnosis determines the degree of differentiation [**5**] and the histopathological form (ductal, lobular, mixed metaplastic tubular) [**6**].

The immunohistochemical tests establish the absence of receptors for estrogen, progesterone, the absence / the presence of HER2 gene and the increased levels of cathepsins D in breast cancer estrogen and the progesterone (-), being a feature that can be targeted in gene therapy [**4**].

According to the immunohistochemical characteristics, the aggressive forms can be classified as: 

● ER(-), PGR(-), HER(-)

● ER(-), PGR(-), HER2(+)

● ER(-), PGR(-), HER2(+++) [**7**].

The immunohistochemical classification depending on HER2 gene is the following:

IHC 0.1 → HER(-)

IHC 3+ → HER(+)

The results of the immunohistochemical tests for the three groups of patients are the following:

● Group A: 37 cases ER(-), PGR(-), HER2(-)

5 cases ER(-), PGR(-), HER2(+)

1 case ER(-), PGR(-), HER2(+++)

● Group B: 36 cases ER(-), PGR(-), HER2(-)

7 cases ER(-), PGR(-), HER2(+)

2 cases ER(-), PGR(-), HER2(+++)

● Group C: 7 cases ER(-), PGR(-), HER2(-)

2 cases ER(-), PGR(-), HER2(+)

Immunohistochemical test results depending on the stage of the disease for the three study groups is the following:

● Group A →stage I: 7 cases ER(-), PGR(-), HER2(-)

1 case ER(-), PGR(-), HER2(+)

 →stage II: 15 cases ER(-), PGR(-), HER2(-) 

 2 cases ER(-), PGR(-), HER2(+)

1 case ER(-), PGR(-), HER2(+++)

→stage III: 4 cases ER(-), PGR(-), HER2(-)

 1 case ER(-), PGR(-), HER2(+)

 →stage IV: 11 cases ER(-), PGR(-), HER2(-)

1 case ER(-), PGR(-), HER2(+) 

● Group B: →stage I: 3 cases ER(-), PGR(-), HER2(-)

→stage II: 12 cases ER(-), PGR(-), HER2(-) 

6 cases ER(-), PGR(-), HER2(+)

1 case ER(-), PGR(-), HER2(+++)

→stage III: 8 cases ER(-), PGR(-), HER2(-)

1 case ER(-), PGR(-), HER2(+++)

 →stage IV: 13 cases ER(-), PGR(-), HER2(-)

1 case ER(-), PGR(-), HER2(+) 

● Group C: 

 →stage II: 2 cases ER(-), PGR(-), HER2(-) 

1 case ER(-), PGR(-), HER2(+)

 →stage III: 2 cases ER(-), PGR(-), HER2(-)

1 case ER(-), PGR(-), HER2(+)

 →stage IV: 3 cases ER(-), PGR(-), HER2(-)

For aggressive forms staging other investigations the following methods were used: breast MRI, CTOs, CT/ MRI/ abdominal and pelvic ultrasound, and lung Rx scintigraphy.

Breast cancer therapy is individual and depends on the stage of the disease, the histopathological form, immunohistochemical tests, which impose the order of neoadjuvant treatment, surgery and adjuvant treatment [**8**].

The following treatment schemes can be used: 

● surgical treatment followed by adjuvant therapy;

● the neoadjuvant treatment + surgical treatment + adjuvant treatment after oncological revaluation.

The neoadjuvant treatment schemes are the following:

● PCT

● PCT + Ac. Anti HER2

The adjuvant treatment schemes can be the following:

● PCT ± Ac. Anti HER2

● PCT + Ac. Anti HER2 + RT

● RT

The surgical treatment applied may be a radical or conservative intervention.

A. Types of mastectomy - radical interventions: 

● Madden – preservation of the two pectoral muscles.

● Patey - rises mammary gland and the anterior fascia of the pectoralis major muscle, axillary lymph nodes tissue, suppressing the smaller pectoral muscle.

● Halsted modified - it removes the mammary gland, muscles, and the axillary lymph nodes tissue in block, when the local situation allows it.

B. Conservative treatment - wide sectorectomy, sectorectomy; associated or cumulated with the avoidance of axillary lymph node stations.

The main treatment for stage I and II is the surgical treatment, preceded or not by neoadjuvant treatment and followed by adjuvant treatment.

The surgical therapy can be conservative (sectorectomy associated with axillary lymphadenectomy) or radical (mastectomy followed or not by breast reconstruction), followed in both cases by adjuvant treatment (PCT ± anti HER 2 ± RT).

The following types of operations were performed for the three batches of patients in the study group:

● Group A - stage I: 4 Madden modified mastectomies; 4 sectorectomies + axillary lymphadenectomy;

 - stage II: 15 Madden mastectomies; 3 sectorectomies + axillary lymphadenectomy;

● Group B - stage I: 3 Madden modified mastectomies;

- stage II: 18 Madden mastectomies; 1 sectorectomy + axillary lymphadenectomy;

● Group C - stage II: 3 Madden modified mastectomies.

For stages III and IV, surgical treatment is preceded by the neoadjuvant treatment PCT ± antibodies anti HER 2 + RT.

Surgical therapy can be: conservative followed by adjuvant treatment (RT breast, infraclavicular, supraclavicular and internal mammary chain + PCT ± anti HER 2) or radically followed by adjuvant treatment (RT thorax, infraclavicular, supraclavicular, internal mammary chain + PCT ± HER2 antibodies); can perform breast reconstruction after radiation therapy ends.

In case there is a negative response after neoadjuvant treatment, it is continued with PCT associated with RT; where the positive response applies the surgery treatment and adjuvant after the previous scheme; in cases in which the answer is negative, individual treatment applies.

The type of surgical procedure performed for the three groups of patients is the following: 

● Group A - stage III: 5 Madden modified mastectomy;

 - stage IV: 8 Madden modified mastectomy: 2 simple mastectomy; 2 simple 

 mastectomy with axillary lymphadenectomy.

● Group B - stage III: 9 Madden modified mastectomy;

- stage IV: 11 Madden modified mastectomy; 1 Madden modified mastectomy 

 + partial resection of the pectoral muscles; 1 simple mastectomy + partial 

resection of the pectoral muscles; 1 sectorectomy + axillary lymphadenectomy.

● Group C - stage III: 3 Madden modified mastectomy;

 - stage IV: 3 Madden modified mastectomy.

Palliative treatment is applied in the following cases: brain metastases, pleural, pericardial, bony, thoracic or biliary obstruction, urethral, impediment fracture, or pathological fracture. The treatment is surgically associated with chemotherapy and radiotherapy. The only postoperative complication that occurred in the study group was lymphorrhagia; the distribution of the 3 groups of patients is the following:

● Group A: st.I – 3 cases; st.II – 5 cases; st.III – 4 cases; st.IV – 10 cases.

● Group B: st.I – 2 cases; st.II – 7 cases; st.III – 7 cases; st.IV – 12 cases.

● Group C: st.II – 2 cases; st.III – 2 cases; st.IV – 2 cases.

There was no presence of a postoperative functional disorder.

Distant metastases on lymphatic way and blood were met in the lungs, bone, liver, SR, ovaries, teguments and CNS.

Distant metastases distribution for the 3 groups of patients is the following:

● Group A

Case 1: Female, 76 years old, st.IV = pT4N1aMi

- Op: Modified radical mastectomy Madden type

- Ex. Hp. = CDI (G3)

- Ex. IHC = ER(-)/ PGR (-)/ HER2(-) 

- High markers

- Pulmonary and hepatic metastases

Case 2: Female, 76 years old, st.IV = pT4N2aMi

- Op: Simple mastectomy with partial lymphadenectomy

- Ex. HP.= CDI (G3)

- Ex. IHC = ER(-)/ PGR (-)/ HER2(+)

- High markers

- Hepatic metastases

● Group B: not a case.

● Group C:

Case 1: Male, 56 years old, st.IV = pT4bN3aMi

- Op: Modified radical mastectomy Madden type

- Ex. HP. = CDI (G3)

- Ex. IHC = ER(-)/ PGR (-)/ HER2(-)

- High markers

- Bony metastases

Case 2: Female, 72 years old, st.IV = pT4bN1aMi

- Op: Modified radical mastectomy Madden type

- Ex. HP. = CDI (G3)

- Ex. IHC = ER(-)/ PGR (-)/ HER2(-) 

- High markers

- Hepatic metastase

The therapeutic conduct is applied in the local recidivism or regionally after the conservative treatment, and it is the following:

- breast recidivism (positive diagnosis is put on the clinical examination and breast MRI) requires total mastectomy ± RT;

- axillary recidivism imposes resection and radiotherapy of the septic over and infraclavicular at thorax level and axillary region;

- supraclavicular recidivism imposes radiotherapy at thorax level, of the septic over and infraclavicular;

- the relapse at the level of the internal mammary ganglionar chain imposes radiotherapy at thorax level, of the fossae over and infraclavicular and internal mammary chain.

- the local relapse after mastectomy imposes radiotherapy at the thorax level. 

3 cases of bilateralism were noticed in the study group (group A: 1 case, group B: 2 cases):

● Group A: Female, 62 years old

→ right breast: 

- Op: Sectorectomy + axillary lymphadenectomy

- Ex. Hp. = CDI (G3)

- Ex. IHC = ER(-)/ PGR (-)/ HER2(-) 

- St.I = pT1N0Mx

- High markers

→left breast: 

- Op: Modified radical mastectomy Madden type

- Ex. Hp. = CDI (G1)

- Ex. IHC = ER(-)/ PGR (-)/ HER2(-) 

- St.I = pT2N1aMx

- High markers

● Lot B: 

Case 1: Female, 57 years old

→right breast: 

- Op: Modified radical mastectomy Madden type

- Ex. Hp. = CDI (G3) + CID

- Ex. IHC = ER(-)/ PGR (-)/ HER2(+++) 

- St.III = pT3NxMx

- High markers

→left breast: 

- Op: Modified radical mastectomy Madden type

- Ex. Hp. = CDI (G2)

- Ex. IHC = ER(+)/ PGR (+)/ HER2(++) 

- St.I = pT1bNxMx

- High markers

Case 2: Female, 62 years old

→right breast:

- Op: Sectorectomy + axillary lymphadenectomy

- Ex. HP. = CDI (G3) + CDIS

- Ex. IHC = ER(-)/ PGR (-)/ HER2(-) 

- St.I = pT1aNxMx

- High markers

→left breast:

- Op: Modified radical mastectomy Madden type

- Ex. HP. = CDI (G3) 

- Ex. IHC = ER(-)/ PGR (-)/ HER2(-) 

- St.II = pT2N0Mx

- High markers

- AHC: the brother with neoplasm lung.

- Postoperative complications: dehiscence wound left breast.

The aggressive breast cancer prognosis depends on demographic characteristics (age, menopausal status), tumoral (lymph node status, tumor size, pathologic type) and biological markers (presence or absence of HER2, the cathepsin D level).

## Conclusion

IHC tests have an important role in determining the neoadjuvant and adjuvant treatment. Female patients who received the neoadjuvant treatment as chemotherapy presented a reducing clinical stage of ggl. Axillary and those who received adjuvant treatment in the form of chemotherapy will benefit from a lower recurrence rate, of the increased long-term survival with both the positive axillary ggl. and those with negative ggl. 

The determination of the overexpression of HER2 receptor suggested a more aggressive and an increased response to chemotherapy (Anthracyclines).
